# 2-(Methyl­sulfin­yl)benzamide

**DOI:** 10.1107/S1600536810046660

**Published:** 2010-11-27

**Authors:** Zhou Yan

**Affiliations:** a College of Food Science and Biotechnology, Zhejiang Gongshang University, Hangzhou 310035, People’s Republic of China

## Abstract

In the crystal of the title compound, C_8_H_9_NO_2_S, synthesized by the oxidation of 2-(methyl­sulfan­yl)benzamide using NaOCl with 2,2,6,6-tetra­methyl­piperidyl-1-oxy (TEMPO) as the catalyst, mol­ecules are linked *via* inter­molecular N—H⋯O_amide_ hydrogen bonds, forming centrosymmetric amide–amide dimers which are extended into a two-dimensional lamellar framework parallel to (100) through amide–sulfinyl N—H⋯O hydrogen bonds. The benzene ring forms a dihedral angle of 25.6 (2)° with the amide group

## Related literature

For general background to sulfoxides, see: Hernández-Torres *et al.* (2008 ▶); Padmanabhan *et al.* (2000 ▶); Nieves & Lang (2002 ▶); Wedel *et al.* (2008 ▶); Melzig *et al.* (2009 ▶); Huang *et al.* (2006 ▶, 2010 ▶). For selective oxidation of sulfides to sulfoxides, see: Huang *et al.* (2006 ▶); Karimi *et al.* (2005 ▶); Kirihara *et al.* (2009 ▶); Ruff *et al.* (2009 ▶). For related structures, see: Kobayashi *et al.* (2003 ▶).
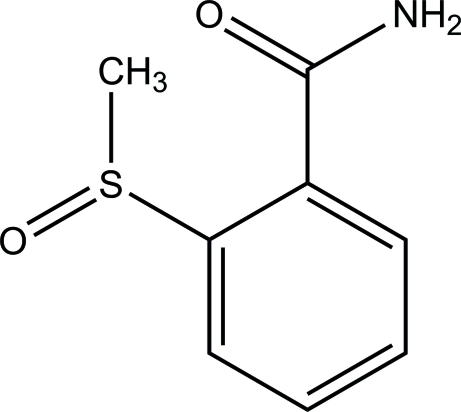


## Experimental

### 

#### Crystal data

C_8_H_9_NO_2_S
*M* *_r_* = 183.22
Monoclinic, 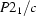

*a* = 11.8497 (5) Å
*b* = 5.0376 (2) Å
*c* = 14.8598 (6) Å
β = 104.856 (4)°
*V* = 857.39 (6) Å^3^
*Z* = 4
Mo *K*α radiation
μ = 0.33 mm^−1^
*T* = 293 K
0.46 × 0.26 × 0.23 mm

#### Data collection

Oxford Diffraction Gemini Ultra CCD-detector diffractometer
Absorption correction: multi-scan (*CrysAlis PRO*; Oxford Diffraction, 2009 ▶) *T*_min_ = 0.901, *T*_max_ = 0.926
3438 measured reflections
1564 independent reflections
1354 reflections with *I* > 2σ(*I*)
*R*_int_ = 0.019

#### Refinement

*R*[*F* ^2^ > 2σ(*F* ^2^)] = 0.030
*wR*(*F* ^2^) = 0.083
*S* = 1.05
1564 reflections
111 parameters
H-atom parameters constrained
Δρ_max_ = 0.25 e Å^−3^
Δρ_min_ = −0.22 e Å^−3^

### 

Data collection: *CrysAlis PRO* (Oxford Diffraction, 2009 ▶); cell refinement: *CrysAlis PRO*; data reduction: *CrysAlis PRO*; program(s) used to solve structure: *SHELXS97* (Sheldrick, 2008 ▶); program(s) used to refine structure: *SHELXL97* (Sheldrick, 2008 ▶); molecular graphics: *OLEX2* (Dolomanov *et al.*, 2009 ▶); software used to prepare material for publication: *OLEX2*.

## Supplementary Material

Crystal structure: contains datablocks global, I. DOI: 10.1107/S1600536810046660/zs2076sup1.cif

Structure factors: contains datablocks I. DOI: 10.1107/S1600536810046660/zs2076Isup2.hkl

Additional supplementary materials:  crystallographic information; 3D view; checkCIF report

## Figures and Tables

**Table 1:** Hydrogen-bond geometry (Å, °)

*D*—H⋯*A*	*D*—H	H⋯*A*	*D*⋯*A*	*D*—H⋯*A*
N1—H1*A*⋯O2^i^	0.86	2.08	2.934 (2)	175
N1—H1*B*⋯O1^ii^	0.86	2.18	2.991 (2)	157

Symmetry codes: (i) 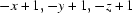; (ii) 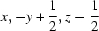.

**Fig. 1.**
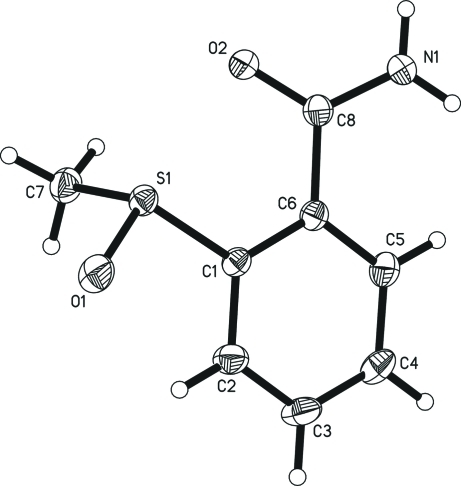
A view of the title compound with showing atom numbering and with displacement ellipsoids drawn at the 30% probability level

**Fig. 2.**
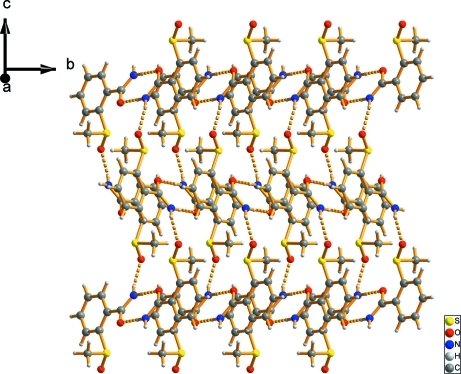
The two-dimensional layered structure of the title compound.

## supplementary crystallographic information

### Comment

Sulfoxides are versatile synthetic intermediates in stereocontrol chemistry (Hernández-Torres *et al.*, 2008). They can be used to prepare chemically and biologically significant molecules, including therapeutic agents such as antiulcer (proton pump inhibitor), antibacterial, antifungal, antiatherosclerotic, antihypertensive, cardiotonic, psychotropic, and vasodilator agents (Padmanabhan *et al.*, 2000; Nieves & Lang, 2002; Wedel *et al.*, 2008; Melzig *et al.*, 2009). The versatility of sulfoxides as organic reagents continually motivate the development of efficient synthesis methods for sulfoxides (Huang *et al.*, 2006; Huang *et al.*, 2010). Although many methods for the synthesis of sulfoxides have been investigated, selective oxidation of sulfides to sulfoxides still remains a challenging task (Karimi *et al.*, 2005; Huang *et al.*, 2006; Kirihara *et al.*, 2009; Ruff *et al.*, 2009). Herein, we report the synthesis and the crystal structure of a sulfoxide, *viz*. the title compound, C_8_H_9_NO_2_S (I). In the crystal structure (Fig. 1), the phenyl ring forms a dihedral angle of 25.6 (2)° with the amide group, similar to that found in benzamide (26.31°) (Kobayashi *et al.*, 2003). The amide groups in (I) give intermolecular N—H···O_amide_ hydrogen-bonding interactions (Table 1) forming centrosymmetric amide–amide dimers which are extended into a two-dimensional lamellar framework parallel to (100), through amide N—H···O_sulfinyl_ hydrogen bonds (Fig. 2).

### Experimental

To a stirred solution of 2-(methylthio)benzamide (167 mg, 1.0 mmol) and the catalyst 2,2,6,6-tetramethylpiperidyl-1-oxy (TEMPO) (1.6 mg, 0.01 mmol) in CH_2_Cl_2_ (8 ml), Bu_4_NBr (16.1 mg, 0.05 mmol) and a saturated aqueous NaHCO_3_ solution (5 ml) containing KBr (11.9 mg, 0.1 mmol) were added. This mixture was cooled to 273 K, a solution of 0.73 *M* NaOCl (0.91 ml, 1.25 mmol) in saturated aqueous NaHCO_3_ was added dropwise over a period of 10 min. The mixture was stirred for a further 1 h at 273 K and for 0.5 h at room temperature. After the organic phase was separated, the aqueous phase was extracted with CH_2_Cl_2_ (3.5 ml) and the organic solution was washed with aqueous brine, dried over anhydrous Na_2_SO_4_ and filtered. The solvent was removed *in vacuo* and the residue was purified by chromatography on silica gel with ethyl acetate/hexane as an eluant to afford the title compound as a white solid (160 mg, 87%). Colorless crystals were obtained by vapor diffusion of hexane into an ethyl acetate solution of (I) over a period of 7 d.

^1^H NMR (400 MHz, CD_3_OD, 295 K) δ (p.p.m.) 8.20–8.18 (1*H*, m), 7.92–7.89 (1*H*, m), 7.85–7.81 (1*H*, m), 7.66–7.62 (1*H*, m), and 2.89 (3*H*, s). ^13^C NMR (400 MHz, CD_3_OD, 295 K) δ (p.p.m.) 168.9, 147.1, 132.4, 131.0, 130.5, 127.7, 123.4, and 43.7.

### Refinement

H atoms bonded to C or N were placed in geometrically calculated positions and were refined using a riding model, with C–H_aromatic_ = 0.93 Å, C—H_methyl_ = 0.96 Å and N—H = 0.86 Å, and with *U*_iso_(H) = 1.2 or 1.5*U*_eq_(C,N).

### Crystal data

**Table:**

C_8_H_9_NO_2_S	*F*(000) = 384
*M**_r_* = 183.22	*D*_x_ = 1.419 Mg m^−^^3^
Monoclinic, *P*2_1_/*c*	Mo *K*α radiation, λ = 0.71073 Å
Hall symbol: -P 2ybc	Cell parameters from 2076 reflections
*a* = 11.8497 (5) Å	θ = 2.8–29.3°
*b* = 5.0376 (2) Å	µ = 0.33 mm^−^^1^
*c* = 14.8598 (6) Å	*T* = 293 K
β = 104.856 (4)°	Block, colorless
*V* = 857.39 (6) Å^3^	0.46 × 0.26 × 0.23 mm
*Z* = 4	

### Data collection

**Table:**

Oxford Diffraction Gemini Ultra CCD-detector diffractometer	1564 independent reflections
Radiation source: fine-focus sealed tube	1354 reflections with *I* > 2σ(*I*)
graphite	*R*_int_ = 0.019
Detector resolution: 10.3592 pixels mm^-1^	θ_max_ = 25.3°, θ_min_ = 2.8°
ω scans	*h* = −14→14
Absorption correction: multi-scan (*CrysAlis PRO*; Oxford Diffraction, 2009)	*k* = −4→6
*T*_min_ = 0.901, *T*_max_ = 0.926	*l* = −14→17
3438 measured reflections	

### Refinement

**Table:**

Refinement on *F*^2^	Secondary atom site location: difference Fourier map
Least-squares matrix: full	Hydrogen site location: inferred from neighbouring sites
*R*[*F*^2^ > 2σ(*F*^2^)] = 0.030	H-atom parameters constrained
*wR*(*F*^2^) = 0.083	*w* = 1/[σ^2^(*F*_o_^2^) + (0.0391*P*)^2^ + 0.2897*P*] where *P* = (*F*_o_^2^ + 2*F*_c_^2^)/3
*S* = 1.05	(Δ/σ)_max_ = 0.001
1564 reflections	Δρ_max_ = 0.25 e Å^−^^3^
111 parameters	Δρ_min_ = −0.22 e Å^−^^3^
0 restraints	Extinction correction: *SHELXL97* (Sheldrick, 2008), Fc^*^=kFc[1+0.001xFc^2^λ^3^/sin(2θ)]^-1/4^
Primary atom site location: structure-invariant direct methods	Extinction coefficient: 0.042 (3)

### Special details

**Table:**

Geometry. All e.s.d.'s (except the e.s.d. in the dihedral angle between two l.s. planes) are estimated using the full covariance matrix. The cell e.s.d.'s are taken into account individually in the estimation of e.s.d.'s in distances, angles and torsion angles; correlations between e.s.d.'s in cell parameters are only used when they are defined by crystal symmetry. An approximate (isotropic) treatment of cell e.s.d.'s is used for estimating e.s.d.'s involving l.s. planes.
Refinement. Refinement of *F*^2^ against ALL reflections. The weighted *R*-factor *wR* and goodness of fit *S* are based on *F*^2^, conventional *R*-factors *R* are based on *F*, with *F* set to zero for negative *F*^2^. The threshold expression of *F*^2^ > σ(*F*^2^) is used only for calculating *R*-factors(gt) *etc*. and is not relevant to the choice of reflections for refinement. *R*-factors based on *F*^2^ are statistically about twice as large as those based on *F*, and *R*- factors based on ALL data will be even larger.

### Fractional atomic coordinates and isotropic or equivalent isotropic displacement parameters (Å^2^)

**Table:**

	*x*	*y*	*z*	*U*_iso_*/*U*_eq_	
S1	0.70440 (4)	0.05668 (9)	0.75213 (3)	0.03131 (18)	
O1	0.78768 (11)	−0.0190 (3)	0.84272 (9)	0.0493 (4)	
O2	0.56496 (11)	0.2416 (3)	0.58587 (9)	0.0443 (4)	
N1	0.64197 (13)	0.4202 (3)	0.47654 (10)	0.0415 (4)	
H1A	0.5843	0.5281	0.4588	0.050*	
H1B	0.6982	0.4221	0.4497	0.050*	
C1	0.77553 (14)	−0.0412 (3)	0.66326 (12)	0.0292 (4)	
C2	0.86744 (15)	−0.2174 (4)	0.68979 (13)	0.0400 (5)	
H2	0.8878	−0.2850	0.7500	0.048*	
C3	0.92910 (16)	−0.2930 (4)	0.62648 (14)	0.0463 (5)	
H3	0.9904	−0.4132	0.6440	0.056*	
C4	0.89971 (16)	−0.1907 (4)	0.53778 (14)	0.0449 (5)	
H4	0.9409	−0.2428	0.4952	0.054*	
C5	0.80940 (16)	−0.0109 (4)	0.51166 (13)	0.0393 (5)	
H5	0.7911	0.0593	0.4518	0.047*	
C6	0.74535 (14)	0.0670 (3)	0.57362 (11)	0.0297 (4)	
C7	0.59562 (16)	−0.1977 (4)	0.73172 (14)	0.0418 (5)	
H7A	0.5457	−0.1791	0.6700	0.063*	
H7C	0.5500	−0.1821	0.7763	0.063*	
H7B	0.6328	−0.3685	0.7379	0.063*	
C8	0.64414 (14)	0.2510 (4)	0.54531 (11)	0.0326 (4)	

### Atomic displacement parameters (Å^2^)

**Table:**

	*U*^11^	*U*^22^	*U*^33^	*U*^12^	*U*^13^	*U*^23^
S1	0.0344 (3)	0.0335 (3)	0.0278 (3)	0.00090 (18)	0.01129 (18)	−0.00260 (18)
O1	0.0443 (8)	0.0777 (11)	0.0256 (7)	0.0052 (7)	0.0086 (6)	−0.0005 (7)
O2	0.0423 (7)	0.0527 (8)	0.0439 (8)	0.0163 (6)	0.0220 (6)	0.0167 (7)
N1	0.0389 (9)	0.0496 (10)	0.0386 (9)	0.0102 (8)	0.0147 (7)	0.0159 (8)
C1	0.0279 (8)	0.0322 (9)	0.0280 (9)	−0.0001 (7)	0.0084 (7)	−0.0029 (7)
C2	0.0363 (10)	0.0476 (12)	0.0350 (10)	0.0094 (9)	0.0069 (8)	0.0010 (9)
C3	0.0362 (10)	0.0525 (13)	0.0506 (12)	0.0148 (9)	0.0118 (9)	−0.0032 (10)
C4	0.0399 (10)	0.0554 (13)	0.0455 (12)	0.0041 (10)	0.0221 (9)	−0.0085 (10)
C5	0.0440 (10)	0.0466 (11)	0.0310 (10)	0.0022 (9)	0.0162 (8)	−0.0005 (8)
C6	0.0300 (9)	0.0317 (9)	0.0282 (9)	−0.0017 (7)	0.0088 (7)	−0.0019 (7)
C7	0.0428 (10)	0.0370 (11)	0.0498 (11)	−0.0030 (9)	0.0195 (9)	0.0003 (9)
C8	0.0350 (9)	0.0359 (10)	0.0269 (9)	0.0009 (8)	0.0080 (7)	−0.0009 (8)

### Geometric parameters (Å, °)

**Table:**

S1—O1	1.5000 (13)	C3—C4	1.374 (3)
S1—C7	1.7875 (19)	C3—H3	0.9300
S1—C1	1.8078 (17)	C4—C5	1.380 (3)
O2—C8	1.239 (2)	C4—H4	0.9300
N1—C8	1.326 (2)	C5—C6	1.391 (2)
N1—H1A	0.8600	C5—H5	0.9300
N1—H1B	0.8600	C6—C8	1.488 (2)
C1—C2	1.382 (3)	C7—H7A	0.9600
C1—C6	1.398 (2)	C7—H7C	0.9600
C2—C3	1.384 (3)	C7—H7B	0.9600
C2—H2	0.9300		
			
O1—S1—C7	104.47 (9)	C5—C4—H4	119.9
O1—S1—C1	105.28 (8)	C4—C5—C6	120.95 (17)
C7—S1—C1	97.56 (8)	C4—C5—H5	119.5
C8—N1—H1A	120.0	C6—C5—H5	119.5
C8—N1—H1B	120.0	C5—C6—C1	118.09 (16)
H1A—N1—H1B	120.0	C5—C6—C8	121.68 (15)
C2—C1—C6	120.83 (16)	C1—C6—C8	120.18 (15)
C2—C1—S1	116.60 (13)	S1—C7—H7A	109.5
C6—C1—S1	122.44 (13)	S1—C7—H7C	109.5
C1—C2—C3	119.85 (17)	H7A—C7—H7C	109.5
C1—C2—H2	120.1	S1—C7—H7B	109.5
C3—C2—H2	120.1	H7A—C7—H7B	109.5
C4—C3—C2	120.01 (18)	H7C—C7—H7B	109.5
C4—C3—H3	120.0	O2—C8—N1	122.13 (16)
C2—C3—H3	120.0	O2—C8—C6	119.62 (15)
C3—C4—C5	120.24 (17)	N1—C8—C6	118.24 (15)
C3—C4—H4	119.9		

### Hydrogen-bond geometry (Å, °)

**Table:**

*D*—H···*A*	*D*—H	H···*A*	*D*···*A*	*D*—H···*A*
N1—H1A···O2^i^	0.86	2.08	2.934 (2)	175
N1—H1B···O1^ii^	0.86	2.18	2.991 (2)	157

Symmetry codes: (i) −*x*+1, −*y*+1, −*z*+1; (ii) *x*, −*y*+1/2, *z*−1/2.
